# Depleting SOX2 improves ischemic stroke via lncRNA PVT1/microRNA-24-3p/STAT3 axis

**DOI:** 10.1186/s10020-021-00346-8

**Published:** 2021-09-14

**Authors:** Zhongjun Chen, Tieping Fan, Xusheng Zhao, Zhichen Zhang

**Affiliations:** 1 Neurological Intervention Department, Dalian Municipal Central Hospital, Dalian, 116033 Liaoning China; 2 Dongfang Hospital of BUCM, Beijing, 100078 China

**Keywords:** Ischemic stroke, Sex determining region Y-box 2, Signal transducer and activator of transcription 3, Long non-coding RNA plasmacytoma variant translocation 1, MicroRNA-24-3p, Oxidative stress

## Abstract

**Objectives:**

Studies have widely explored in the filed of ischemic stroke (IS) with their focus on transcription factors. However, few studies have pivoted on sex determining region Y-box 2 (SOX2) in IS. Thus, this study is launched to figure out the mechanisms of SOX2 in IS.

**Methods:**

Rat middle cerebral artery occlusion (MCAO) was established as a stroke model. MCAO rats were injected with depleted SOX2 or long non-coding RNA plasmacytoma variant translocation 1 (PVT1) to explore their roles in neurological deficits, cerebral water content, neuron survival, apoptosis and oxidative stress. The relationship among SOX2, PVT1, microRNA (miR)-24-3p and signal transducer and activator of transcription 3 (STAT3) was verified by a series of experiments.

**Results:**

SOX2, PVT1 and STAT3 were highly expressed while miR-24-3p was poorly expressed in cerebral cortex tissues of MCAO rats. Depleted SOX2 or PVT1 alleviated brain injury in MCAO rats as reflected by neuronal apoptosis and oxidative stress restriction, brain water content reduction, and neurological deficit and neuron survival improvements. Overexpression of PVT1 functioned oppositely. Restored miR-24-3p abolished PVT1 overexpression-induced brain injury in MCAO rats. SOX2 directly promoted PVT1 expression and further increased STAT3 by sponging miR-24-3p.

**Conclusion:**

This study presents that depleting SOX2 improves IS via PVT1/miR-24-3p/STAT3 axis which may broaden our knowledge about the mechanisms of SOX2/PVT1/miR-24-3p/STAT3 axis and provide a reference of therapy for IS.

## Introduction

Ischemic stroke (IS) is generally considered as a leading cause of death and disability around the world (Liu et al. 2015). The occurrence of IS may result from major atherosclerotic disease, cardiogenic or arterial-to-arterial embolism, small vessel or penetrating arterial disease, hypercoagulable diseases, non-atherosclerotic vascular disease, or infarction-induced factors (Ashjazadeh et al. 2013). As to the treatment of acute IS, tissue plasminogen activator accounts for the only drug approved by Food and Drug Administration (Wang et al. 2015). However, considering the high incidence and mortality (Zhang et al. 2016), it is a pressing urgency to bring about novel therapies for IS.

Sex determining region Y-box 2 (SOX2) is a transcription factor that regulates self-renewal and differentiation of embryonic stem cells (Li et al. 2012). To our best knowledge, it is recorded that SOX2 expression is elevated after hypoxia in cerebral ischemia (Han et al. 2017). Also, SOX2 is found to be highly expressed in the hippocampus after traumatic brain injury (Gu et al. 2016). MicroRNAs (miRs) function in the processes of neurodegenerative diseases and certain neurological diseases including IS (Eyileten et al. 2018). Similarly, miR-24-3p is correlated with angiogenic factors that have been associated with recurrent ischemic events in intracranial atherosclerotic disease (Jiang et al. 2019). Specifically, miR-24-3p is regarded to be involved in the process of neuron cell damage under oxygen-glucose deprivation/reperfusion conditions (Di et al. 2021). As a subfamily of long non-coding RNA, plasmacytoma variant translocation 1 (PVT1) is often linked to oncogenesis of malignant cancers including a brain tumor, glioma (Fang and Huang 2019). A former study has revealed that PVT1 inhibition can be applied to improve spatial learning and memory, and reduce neuronal loss and neuronal apoptosis in epilepsy (Zhao et al. 2019). Signal transducer and activator of transcription 3 (STAT3) pathway is involved in modulation of microglia/macrophage polarization toward anti-inflammatory phenotype in IS (Liu et al. 2019). Also, inhibition of janus kinase (JAK)/STAT3 pathway exerts a functional role in oxidative stress-induced neuronal injury in mice with IS (Li et al. 2020). As mentioned formerly, there is a transcriptional factor binding site between SOX2 and PVT1 promoter and PVT1 can be activated by SOX2 (Wang et al. 2017). Interestingly, circular (circ) RNA PVT1 elevates STAT3 expression via sponging miR-125b in oral squamous cell carcinoma (He et al. 2019).

Taken together, the more in-depth translation of SOX2 into clinical application needs more logical explorations in IS. Thus, this study is carried out to make some advances in the mechanisms and participation of SOX2, PVT1, STAT3 and miR-24-3p in IS.

## Materials and methods

### Ethics statement

This experiment was approved by the animal ethics committee of Dongfang hospital of BUCM. Great efforts have been made to relieve pain for animals.

### Rat middle cerebral artery occlusion (MCAO) model establishment

Healthy adult male Sprague-Dawley rats of clean grade (250–300 g, the Center for Experimental Animals at China Medical University, Shenyang, China) had free access to food and pure water (12-h day and night cycle, 23 ± 2 °C, 55 ± 5% humidity). The rats were acclimated to the experimental environment for 3 days before experiments.

Model establishment (Mehta et al. 2015): The rats were anesthetized with intraperitoneal injection of 1% pentobarbital sodium (Sigma, Santa Clara, CA, USA). The rats were fixed in a supine position and a longitudinal midline incision was made on the neck to expose and bluntly separate the left common carotid artery. The dismal end of the external carotid artery was ligated and cut off with an artery occlusion while the stump of it was threaded to prevent blood flow, and the artery occlusion was released. A suture was inserted into the internal carotid artery from the stump of the external carotid artery to the site 18 mm from the bifurcation of the common carotid artery. The rat MCAO model was successfully established with the blood flow value below 30% of the baseline detected by a transcranial laser Doppler flowmeter. The anterior neck incision was sutured and disinfected. The rats in the sham group were treated as same as those in the MCAO group, except that no suture was inserted.

### Rat grouping and treatment

Rats were assigned into nine groups (n = 13). The rats in the sham group and the MCAO group were treated in the aforementioned model establishment way while rats in the other seven groups were injected with an shRNA targeting SOX2 lentiviral vector (sh-SOX2), sh-SOX2 NC lentiviral vector (sh-NC), an shRNA targeting PVT1 lentiviral vector (sh-PVT1), sh-PVT1 NC lentiviral vector (sh-CTR), lentiviral vector overexpressing PVT1 (Lenti-PVT1), lentiviral vector NC (Lenti-NC), or lentiviral vector overexpressing PVT1 + lentiviral vector overexpressing miR-24-3p (Lenti-miR-24-3p) into the left lateral ventricle 24 h before modeling (injection in 5 min with 3.0 mm of depth). The above lentiviral vectors were prepared by GenePharma (Shanghai, China).

### Forelimb foot fault placing test

Five rats in each group were randomly selected for motor behavioral evaluation 7 days after MCAO. Forelimb foot fault placing test was performed for forelimb movement ability detection. A grid plate of 10 × 110 cm^2^ (grid diameter 0.1 cm, grid hole area 3 × 3 cm^2^) was placed 100 cm above the ground. During the test, the rats were stimulated to pass through the grid plate within 1 min. The times of the forelimb stepping into the grid hole on the contralateral side of the lesion (the forelimb misplacement times) were counted. The forelimb misplacement times of rats passing 100 cm on the grid plate were recorded. Rats were scored for 0 point without any forelimb misplacement; 1 point for forelimb misplacement ≤ 1 time; 2 points for forelimb misplacement > 1 time; 3 points for forelimb misplacement ≥ 2 times; 4 points for forelimb misplacement ≥ 3 times; 5 points for disability to pass through the grid plate.

#### Parallel bar test

Two parallel wooden bars (1 cm in diameter and 110 cm in length) with a distance of 2.5 cm were connected with the platform. Rats were triggered to crawl from one end of the platform to the other. During the test, the crawling time, distance, and errors (the times that both hind limbs of rats placing on the same bar, the times of the hind limbs of rats slipping, and the times of rats falling from the parallel bars) were recorded. The errors of rats crawling 100 cm of the bars in 1 min were counted and rats were scored for 0 point without any errors; 1 point for errors ≤ 1 time; 2 points for errors > 1 time; 3 points for errors ≥ 2 times; 4 points for errors ≥ 3 times; 5 points for ≥ 6 times.

#### Rope climbing test

A rope (1.5 cm in diameter) was hang from a platform (100 cm in height, 15 cm in length, 50 cm in width). Each rat was trained 1 days before test to ensure that it could climb up to the platform. During the test, rats were stimulated to climb up the rope. The time required for rats to climb the platform and the number of stimuli required during the climbing were recorded. Rats were scored for 0 point for climbing to the platform within 10 s without stimulation; 1 point for climbing to the platform within 15 s without stimulation; 2 points for climbing to the platform within 30 s with stimulation < 5 times; 3 points for climbing to the platform within 60 s with stimulation < 5 times; 4 points for climbing to the platform within 60 s with stimulation > 5 times or climbing time > 60 s and stimulation < 5 times; 5 points for disability to climb or even grasp the rope.

### Neurological examination

Eight rats in each group were subjected to neurological examination 24 h after MCAO. Rats were scored for their neurological deficit with 0 point for rats without deficit; 1 point for incomplete contralateral forelimb extension, 2 points for disability of contralateral forelimb extension, 3 points for slight contralateral rotation, 4 points for severe contralateral rotation and 5 points for contralateral tumbling.

### Brain water content determination

At 24 h post MCAO, the cerebral cortex (about 5 mm) on the lesion side of the rat was weighed by an electronic balance (accuracy of 0.001 g), and baked at 100 °C for 48 h, namely dry weight. Brain water content was calculated according to Elliott’s formula: brain water content (%) = (wet weight − dry weight)/wet weight × 100%.

### HE staining

At 24 h post MCAO, the cerebral cortex tissue of the ischemic side of the rat was routinely prepared for paraffin sections. The slices were dewaxed, hydrated and stained by hematoxylin solution for 3–5 min, which was followed by differentiation in dilute hydrochloric acid and ammonia water and dehydration in 70% and 90% alcohol for 10 min each. Subsequently, the slices were stained in eosin for 2–3 min, dehydrated with absolute alcohol and permeabilized with xylene. The dried and permeabilized slices were sealed with neutral resin and observed under a light microscope with nucleus in blue and cytoplasm in red.

### Nissl staining

The paraffin slices were baked at 55 °C for 30 min, dewaxed in xylene for 5–10 min, soaked in absolute ethanol, 90% ethanol, and 70% ethanol for 2 min each, and rinsed with distilled water for 5 min. The preheated Nissl staining solution (Beyotime Biotechnology Co., Shanghai, China) at 35 °C was applied for staining for 10 min and the slices were rinsed with distilled water and 90% alcohol, dehydrated with 95% ethanol for 2 min which was followed by permeabilization by xylene and baking at 37 °C. After that, the sealed slices in neutral resin were observed under a light microscope. Normal neurons had clear nuclei and nucleoli, distinguished axons and dendrites and small Nissl bodies. Around the nucleus, Nissl bodies were manifested in large and lumpy granules and darkly stained while they became smaller near the edges and lightly stained.

### TUNEL staining

According to the instructions of the TUNEL kit (Wuhan Boster Biological Technology Co., Ltd., Hubei, China), the paraffin slices were treated with xylene, 100%, 95%, 80%, and 70% alcohol successively. After that, the slices were immersed in 4% paraformaldehyde for 30 min, incubated with 0.1% Triton X-100 sodium citrate buffer for 20 min, and added with TUNEL reaction mixture for 1–1.5 h. Subsequently, the slices were added with peroxidase reaction solution for 30 min which was followed by diaminobenzidine (DAB) development for 5–10 min. Next, the slices were counterstained with hematoxylin solution, dehydrated, permeabilized, and sealed in neutral gum for observation under a light microscope. Cells with brownish-yellow nucleus were TUNEL-positive apoptotic cells. Observed with five different fields under high magnification, TUNEL-positive cells were counted and the apoptosis index (AI [%]) was calculated: (number of apoptotic cells/total number of cells) × 100%.

### Immunohistochemistry

Paraffin sections were serially sliced to 4 µm and baked at 60 °C for 1 h. Then, the sections were dewaxed and dehydrated with xylene I and II, and gradient alcohol, and soaked in 3% hydrogen peroxide. Subsequently, the sections were blocked with 10% goat serum, probed with primary antibodies SOX2 (1:150, ab97959, Abcam, USA) and STAT3 (1:150, ab119352, Abcam), and with biotin-labeled secondary antibody IgG (ab97051, 1:2000, Abcam). After development with DAB (DA1010, Solarbio, Beijing, China), the sections were dyed with hematoxylin (H8070, solarbio), dehydrated with gradient alcohol, permeabilized with xylene and mounted with neutral gum. PBS was used as a negative control instead of primary antibody. The final result was scored by two double-blindly. Five high-power fields were randomly selected under an optical microscope (CX41-12C02, Olympus, Japan), and the percentage of positive cells with brown particles were calculated.

### Oxidative stress detection

Twenty-four hours after MCAO, the cerebral cortex tissue of the ischemic side of the rat was quickly stored at – 80 ℃ for examination of the levels of malondialdehyde (MDA), superoxide dismutase (SOD) and glutathione (GSH). The frozen tissues were thawed, rinsed with 4 °C normal saline, dried with filter paper, and weighed. Then, the tissues were mixed with normal saline at 1:9 and homogenized in ice water bath which was followed by centrifugation (2500 r/min, 10 min). After that, the supernatant was diluted with normal saline at 1:5, and 50 μL of the diluted supernatant was applied to detection of SOD, MDA and GSH by the instructions of their detection kits (NanJing JianCheng Bioengineering Institute, Nanjing, China).

### Reverse transcription quantitative polymerase chain reaction (RT-qPCR)

Total RNA was extracted from cerebral cortex tissue on the ischemic side after 24 h of MCAO using Trizol reagent (Invitrogen, Carlsbad, CA, USA). cDNA was obtained by the kit (ABM, Richmond, BC, Canada), and target gene expression was determined using an ABI 7500 sequence detection system (Applied Biosystems, CA, USA). U6 was applied as an internal control for miR-24-3p while glyceraldehyde-3-phosphate dehydrogenase (GAPDH) for SOX2, PVT1, and STAT3. The results were analyzed by the relative quantitative 2^−ΔΔCT^ method. The primers used in the experiments were shown in Table 1.

**Table 1:** Primer sequence

Gene	Primer sequence
miR-24-3p	Forward: 5ʹ-GCCGAGTGGCTCAGTTCAGC-3ʹ
	Reverse: 5ʹ-CTCAACTGGTGTCGTGGA-3ʹ
U6	Forward: 5ʹ-CGCTTCGGCAGCACATATAC-3ʹ
	Reverse: 5ʹ-AAATATGGAACGCTTCACGA-3ʹ
SOX2	Forward: 5ʹ-AGAACCCCAAGATGCACAAC-3ʹ
	Reverse: 5ʹ-ATGTAGGTCTGCGAGCTGGT-3ʹ
PVT1	Forward: 5ʹ-GCCCCTTCTATGGGAATCACTA-3ʹ
	Reverse: 5ʹ-GGGGCAGAGATGAAATCGTAAT-3ʹ
STAT3	Forward: 5ʹ-CACCCATAGTGAGCCCTTGGA-3ʹ
	Reverse: 5ʹ-TGAGTGCAGTGACCAGGACAGA-3ʹ
GAPDH	Forward: 5ʹ-TGACGTGCCGCCTGGAGAAAC-3ʹ
	Reverse: 5ʹ-CCGGCATCGAAGGTGGAAGAG-3ʹ

*miR-24-3p* microRNA-24-3p, *SOX2* Sex determining region Y-box 2, *PVT1* Long non-coding plasmacytoma variant translocation 1, *STAT3* Signal transducer and activator of transcription 3, *GAPDH* Glyceraldehyde-3-phosphate dehydrogenase

### Western blot analysis

Total protein in cerebral cortex tissue on the ischemic side after 24 h of MCAO was extracted and lysed by radio immunoprecipitation assay buffer (Beyotime, Haimen, China). The protein concentration was determined by the bicinchoninic acid protein assay kit (Beyotime). The protein lysate was added to the 12% sodium dodecyl sulfate polyacrylamide gel per well. The protein was then transferred to a polyvinylidene fluoride (PVDF, Millipore, Boston, Massachusetts, USA) membrane and blocked with 5% skim milk powder in tris-buffered saline with Tween 20 (1% Tween-20) for 2 h. The membrane was probed with primary antibodies STAT3 (1:1000), SOX2 (1:2000, Abcam) or GAPDH (1:1000, Cell Signaling Technology, Beverly, MA, USA) overnight, and re-probed with the horseradish peroxidase-linked secondary antibody for 2 h. The membrane was developed by an enhanced chemiluminescence developer (Thermo Fisher Scientific, MA, USA), imaged by BIO-RAD gel imager and evaluated by Image Lab software. The ratio of the gray value of the target protein band to the GAPDH band was calculated (Wu et al. 2017).

### Dual luciferase reporter gene assay

Luciferase reporters (Promega, WI, USA) were constructed with wild type (Wt) and mutant type (Mut), respectively. Firefly luciferase represents the main reporter gene that monitors the binding of proteins/miRNAs to cloned target sequences. Renilla luciferase is regarded as a standardized control reporter gene. Luciferase reporters and miR-24-3p mimic or mimic NC were co-transfected into HEK293 cells or rat neurons-cortical (RN-c, R1520, Shanghai Zhongqiao Xinzhou Biotechnology Co., Ltd., Shanghai, China) cells through Lipofectamine 3000 reagent. After 48 h, the luciferase activity was measured by the dual luciferase reporter gene detection kit (Promega).

### RNA-pull down assay

RNA pull-down was allowed to determine the interaction between PVT1 and miR-24-3p. HEK293 cells or RN-c cells were added with lysis buffer containing protease inhibitors (Solarbio, Beijing, China), mixed with biotin-labeled lncRNA probe sequence, and incubated with M-280 streptavidin-coated magnetic beads (Invitrogen). After washing the beads with ice-cold lysis buffer, low-salt buffer and high-salt buffer in sequence, the bound RNA was collected using Trizol reagent (Invitrogen), and quantified by RT-qPCR.

### RNA immunoprecipitation (RIP) assay

The Magna RIP Kit (Millipore) was employed to determine the relationship between PVT1 and miR-24-3p using anti-AGO2 and IgG (Millipore). Co-precipitated RNA was analyzed by RT-qPCR.

### Chromatin immunoprecipitation (ChIP) assay

HEK-293 cells or RN-c cells were fixed with formaldehyde using an EZ-ChIP™ kit (Millipore) and incubated with glycine. The anti-SOX2 antibody and immunoglobulin (IgG, Abcam) were used to precipitate the cross-linked protein-DNA complex. The precipitated DNA was analyzed using RT-qPCR.

### Statistical analysis

All data were analyzed by GraphPad Prism 6.0 software (GraphPad Software, CA, USA) and SPSS 20.0 software (IBM, NY, USA) statistical software. Data were expressed as mean ± standard deviation and compared by Student’s t-test or one-way analysis of variance (ANOVA). *P* < 0.05 was considered statistically significant.

## Results

### SOX2, PVT1 and STAT3 are highly expressed while miR-24-3p is poorly expressed in brain tissues in MCAO rats

**Fig. 1:**
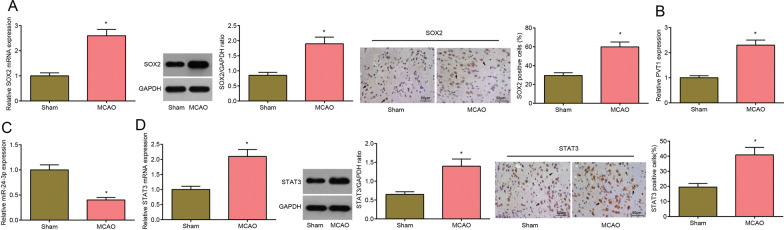
SOX2, PVT1 and STAT3 are highly expressed while miR-24-3p is poorly expressed in cerebral cortex tissues in MCAO rats. **A** SOX2 expression in cerebral cortex tissues of MCAO rats (× 200; scale bar = 50 μm; arrows indicated positive cells by immunohistochemical staining); **B** PVT1 expression in cerebral cortex tissues of MCAO rats; **C** miR-24-3p expression in cerebral cortex tissues of MCAO rats; **D** STAT3 expression in cerebral cortex tissues of MCAO rats (× 200; scale bar = 50 μm; arrows indicated positive cells by immunohistochemical staining); n = 8 rats per group; *P < 0.05 compared with the sham group

RT-qPCR, Western blot and immunohistochemistry were used to detect SOX2, PVT1, miR-24-3p, and STAT3 expression in cerebral cortex in each group. The results demonstrated that relative to the sham group, SOX2, PVT1, and STAT3 expression elevated and miR-24-3p expression decreased in the MCAO group (all *P* < 0.05) (Fig. 1A–D).

### Inhibited SOX2 or PVT1 upregulates miR-24-3p expression and downregulate STAT3 expression in MCAO rats

**Fig. 2:**
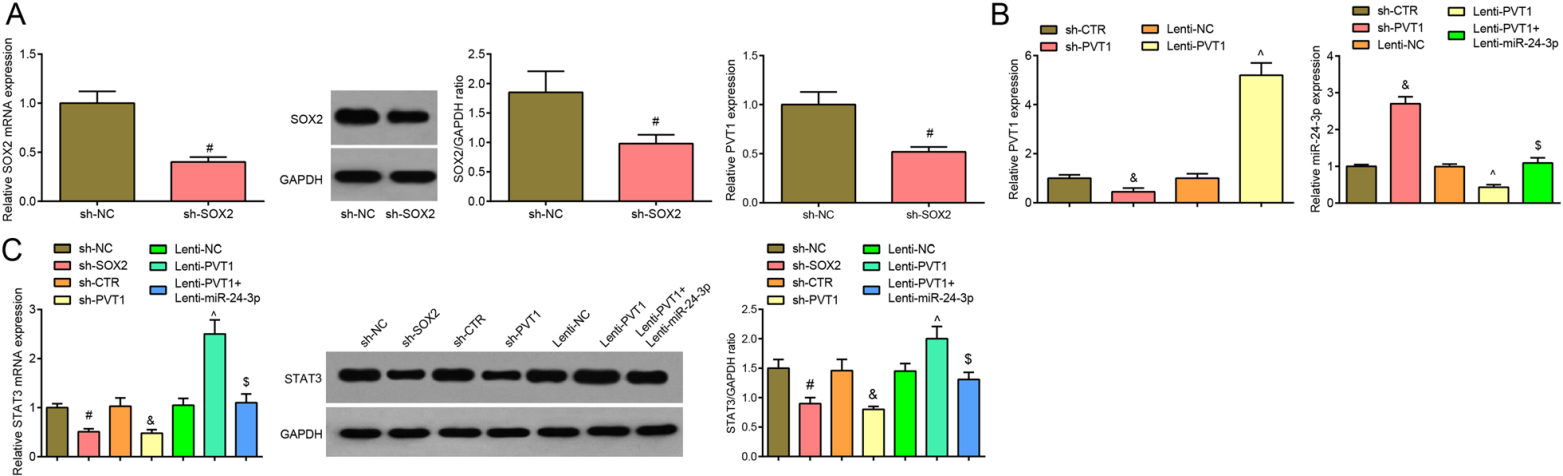
Inhibited SOX2 or PVT1 upregulates miR-24-3p expression and downregulates STAT3 expression in MCAO rats. **A** SOX2, PVT1 expression in MCAO rats cerebral cortex tissues in each group after inhibition of SOX2; **B** PVT1, miR-24-3p expression in MCAO rats cerebral cortex tissues in each group after inhibition of PVT1; **C** STAT3 expression in MCAO rats cerebral cortex tissues in each group after inhibition of SOX2 or PVT1; n = 8 rats per group; ^#^*P* < 0.05 compared with the sh-NC group; ^&^*P* < 0.05 compared with the sh-CTR; ^^^*P* < 0.05 compared with the Lenti-NC group; ^$^*P* < 0.05 compared with the Lenti-PVT1 group

It was indicated by RT-qPCR and Western blot analysis (Fig. 2A–C) that the sh-SOX2 group showed reduced PVT1 and STAT3 expression and increased miR-24-3p expression versus the sh-NC group (all *P* < 0.05); by contrast to the sh-CTR group, the sh-PVT1 group presented decreased STAT3 expression and elevated miR-24-3p expression (both *P* < 0.05); with respect to the Lenti-NC group, the Lenti-PVT1 group exhibited increased STAT3 expression and decreased miR-24-3p expression (both *P* < 0.05). Versus the Lenti-PVT1 group, STAT3 expression declined and miR-24-3p expression increased in the Lenti-PVT1 + Lenti-miR-24-3p group (both *P* < 0.05).

### Depleted SOX2 or PVT1 alleviates brain injury in MCAO rats

**Fig. 3:**
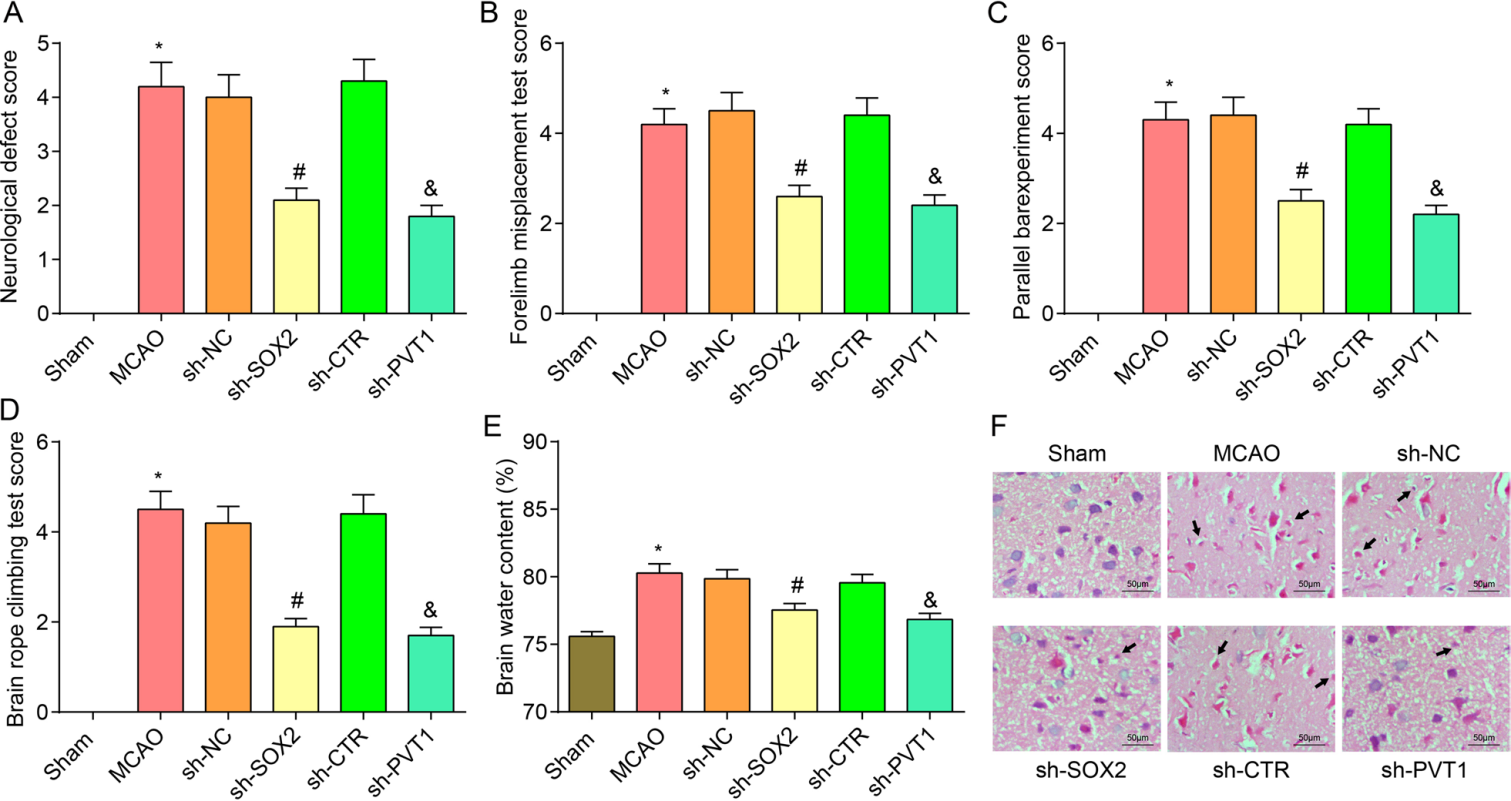
Depleted SOX2 or PVT1 attenuates neurological deficits, motor behavioral and pathological damage in MCAO rats. **A** Neurological defect scores of rats in each group after inhibition of SOX2 or PVT1 (n = 8 rats per group); **B** Forelimb foot fault placing test scores of rats in each group after inhibition of SOX2 or PVT1 (n = 5 rats per group); **C** Parallel bar experiment scores of rats in each group after inhibition of SOX2 or PVT1 (n = 5 rats per group); **D** Rope climbing test scores of rats in each group after inhibition of SOX2 or PVT1 (n = 5 rats per group); **E** Brain water content of rats in each group after inhibition of SOX2 or PVT1 (n = 8 rats per group); **F** HE staining of rat brain injury in each group after inhibition of SOX2 or PVT1 (× 200; scale bar = 50 μm, n = 8 rats per group); arrow indicated that the nucleus of neurons appears with pyknosis; *P < 0.05 compared with the sham group; ^#^*P* < 0.05 compared with the sh-NC group; ^&^*P* < 0.05 compared with the sh-CTR group

The results of neurological defect score illustrated that versus the sham group, the MCAO group had severer neurological deficits (*P* < 0.05). In comparison with the sh-NC group and the sh-CTR group, the sh-SOX2 group and the sh-PVT1 group manifested improved neurological deficits (both *P* < 0.05) (Fig. 3A).

The findings of behavior tests presented that with respect to the sham group, higher forelimb misplacement score, parallel bar experiment score, and rope climbing test score were found in the MCAO group. With contrast to the sh-NC group and the sh-CTR group, decreased behavioral test scores were characterized in the sh-SOX2 group and the sh-PVT1 group, respectively (all *P* < 0.05) (Fig. 3B–D).

Brain water content results depicted that brain water content increased in the MCAO group in comparison with the sham group while decreased in the sh-SOX2 group and the sh-PVT1 group with the sh-NC group and the sh-CTR group by contrast (both *P* < 0.05) (Fig. 3E).

HE staining results indicated that (Fig. 3F) ischemic cerebral cortex in the sham group demonstrated complete and clear structure without intercellular substance edema and cells were arranged orderly and stained uniformly. The contused brain tissues in the MCAO group, the sh-NC group, and the sh-CTR group exhibited liquefaction and necrosis, disordered cell arrangement, edema around the contusion, nucleus shrinkage and even dissolution, and inflammatory cell infiltration. The sh-SOX2 and the sh-PVT1 groups showed reduced local bleeding, and improved edema around the contusion.

**Fig. 4:**
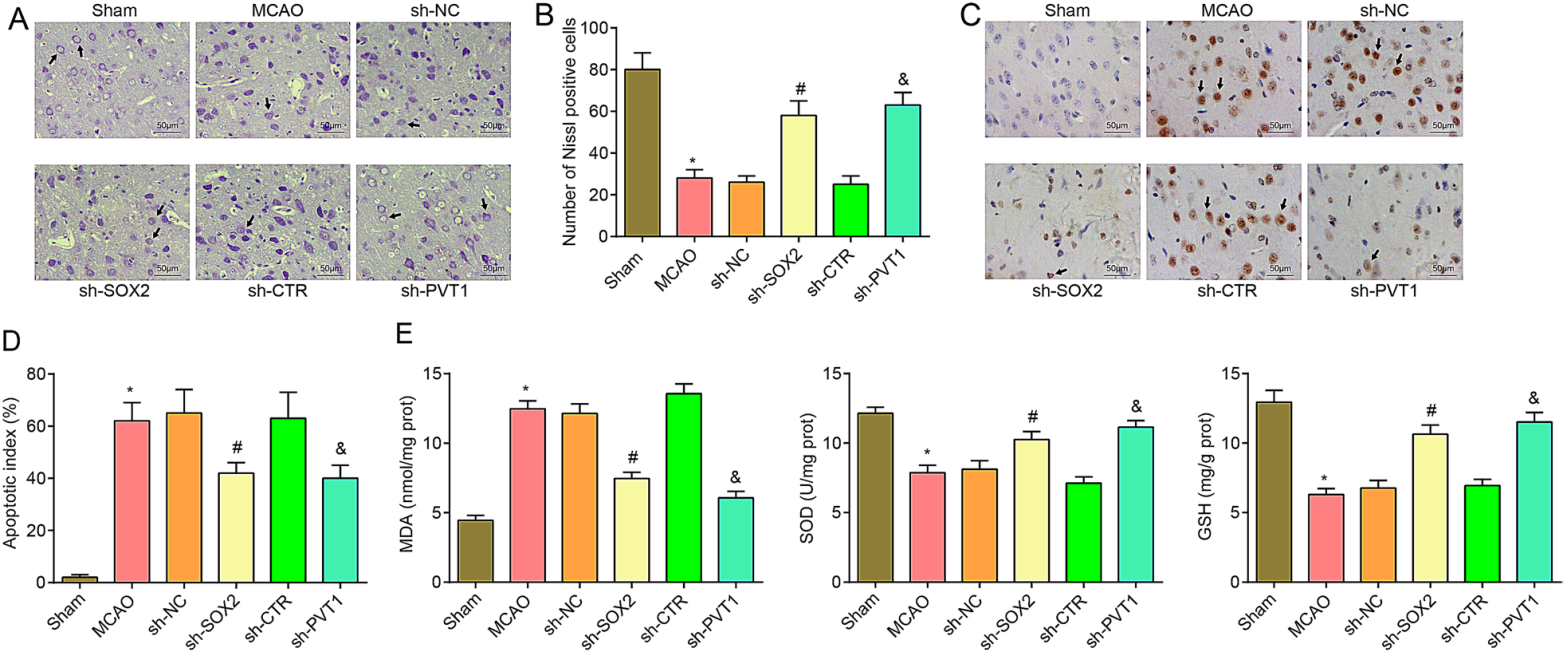
Depleted SOX2 or PVT1 increases neuronal survival, reduces neuronal apoptosis and oxidative stress in the cerebral cortex of MCAO rats. **A**/**B** Nissl staining of neuron survival in each group after inhibition of SOX2 or PVT1 (× 200; scale bar = 50 μm, n = 8 rats per group); arrows indicated Nissl bodies; **C**/**D** TUNEL staining of neuronal apoptosis in each group after inhibition of SOX2 or PVT1 (× 200; scale bar = 50 μm, n = 8 rats per group); arrows indicated TUNEL-positive cells; **E** Oxidative stress indicators in the cortex of rats in each group after inhibition of SOX2 or PVT1 (n = 8 rats per group); *P < 0.05 compared with the sham group; ^#^*P* < 0.05 compared with the sh-NC group; ^&^*P* < 0.05 compared with the sh-CTR group

Nissl staining results demonstrated that by comparison with the sham group, the MCAO group manifested largely dissolved or disappeared Nissl bodies, lightly stained cytoplasm, reduced number of Nissl bodies in the MCAO group (*P* < 0.05). With respect to the sh-NC group and the sh-CTR group, the sh-SOX2 group and the sh-PVT1 group showed with darkly stained cytoplasm, increased number of Nissl bodies (both *P* < 0.05) (Fig. 4A, B).

TUNEL staining indicated that by comparison with the sham group, increased number of TUNEL-positive neurons was detected in the MCAO group (*P* < 0.05); versus the sh-NC group and the sh-CTR group, respectively, the TUNEL-positive neurons was suppressed in the sh-SOX2 group and the sh-PVT1 group (both *P* < 0.05) (Fig. 4C, D).

Detection of the oxidative stress indicators in the cerebral cortex outlined that by comparison with the sham group, MDA content elevated, and SOD and GSH activities impaired in the MCAO group (all *P* < 0.05). In relation to the sh-NC group and the sh-CTR group, MDA decreased while SOD and GSH activities reinforced in the sh-SOX2 group and the sh-PVT1 group (all *P* < 0.05) (Fig. 4E).

### Restored miR-24-3p abolishes PVT1 overexpression-induced brain injury in MCAO rats

**Fig. 5:**
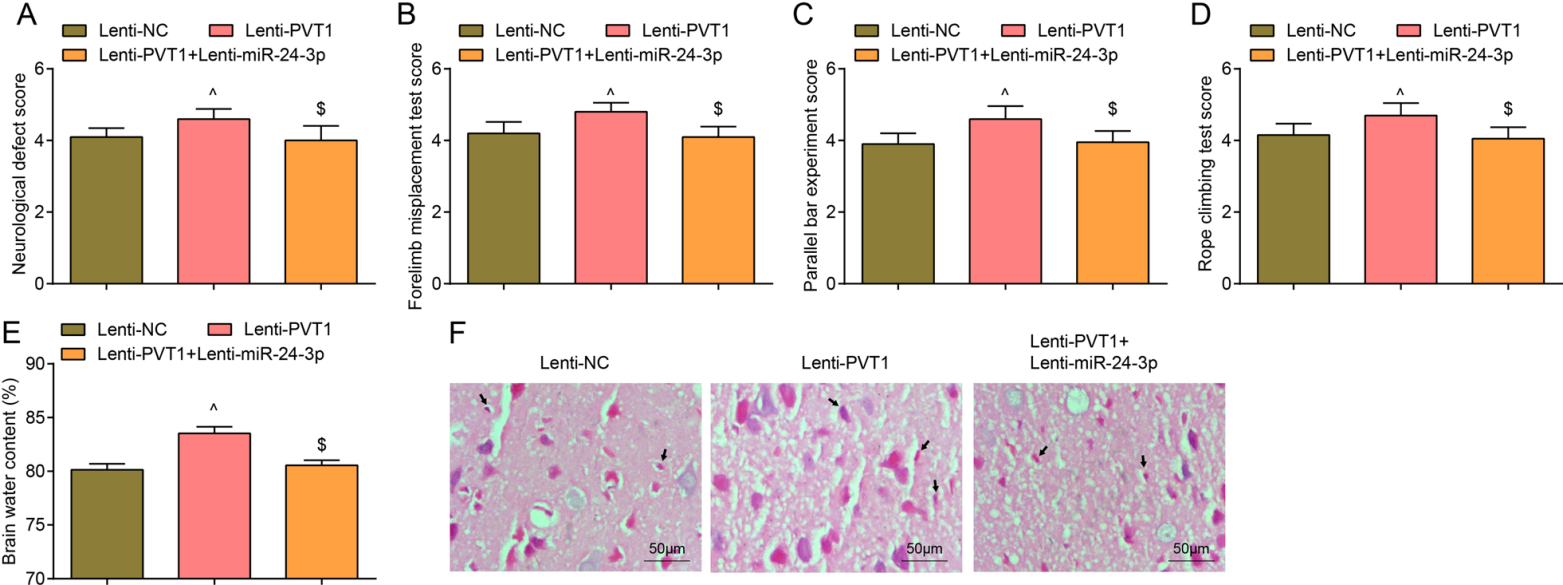
Restored miR-24-3p abolishes PVT1 overexpression-induced neurological impairment, motor behavioral disorder and pathological damage in MCAO rats. **A** Neurological defect scores of rats in each group after PVT1 overexpression and miR-24-3p up-regulation (n = 8 rats per group); **B** Forelimb foot fault placing test scores of rats in each group after PVT1 overexpression and miR-24-3p up-regulation (n = 5 rats per group); **C** Parallel bar experiment scores of rats in each group after PVT1 overexpression and miR-24-3p up-regulation (n = 5 rats per group); **D** Rope climbing test scores of rats in each group after PVT1 overexpression and miR-24-3p up-regulation (n = 5 rats per group); **E** Brain water content of rats in each group after PVT1 overexpression and miR-24-3p up-regulation (n = 8 rats per group); **F** HE staining of rat brain injury in each group after PVT1 overexpression and miR-24-3p up-regulation (× 200; scale bar = 50 μm, n = 8 rats per group; arrow indicated that the nucleus of neurons appears with pyknosis); ^^^*P* < 0.05 compared with the Lenti-NC group; ^$^*P* < 0.05 compared with the Lenti-PVT1 group

The results of neurological defect score in MCAO rats illustrated that relative to the Lenti-NC group, the neurological deficits were worsened in the Lenti-PVT1 group (*P* < 0.05). Versus the Lenti-PVT1 group, the degree of neurological deficits was improved in the Lenti-PVT1 + Lenti-miR-24-3p group (*P* < 0.05) (Fig. 5A).

Behavior tests reflected that versus the Lenti-NC group, the scores behavior tests were higher in the Lenti-PVT1 group (*P* < 0.05). In contrast to the Lenti-PVT1 group, the scores of behavior tests were decreased in the Lenti-PVT1 + Lenti-miR-24-3p group (*P* < 0.05) (Fig. 5B–D).

Brain water content results indicated that brain water content increased in the Lenti-PVT1 group by comparison with the Lenti-NC group while decreased in the Lenti-PVT1 + Lenti-miR-24-3p group in relation to the Lenti-PVT1 group (all *P* < 0.05) (Fig. 5E).

HE staining results pictured (Fig. 5F) contusion brain tissue liquefaction and necrosis, disordered structure, edema around the contusion, constriction and even dissolution of nucleus, and inflammatory cell infiltration in the Lenti-NC group and the Lenti-PVT1 + Lenti-miR-24-3p group. The Lenti-PVT1 group suffered from extensive necrosis of contused brain tissues, exacerbated edema, and severe inflammatory infiltration.

**Fig. 6:**
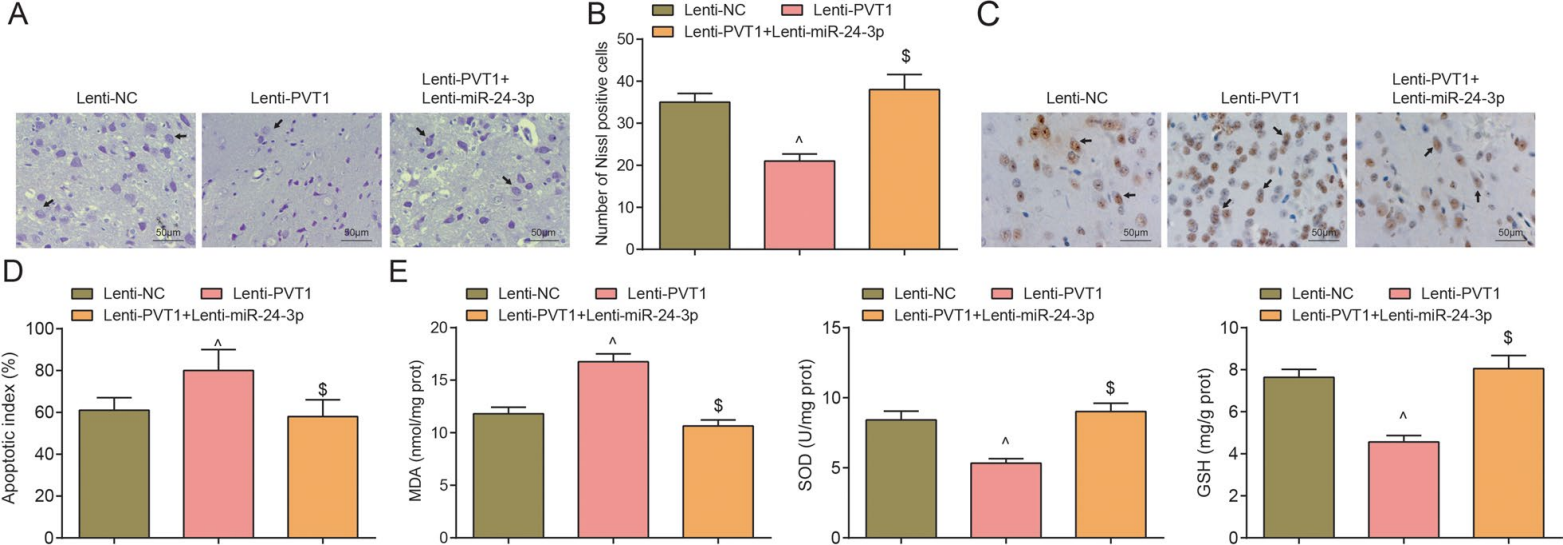
Restored miR-24-3p abolishes PVT1 overexpression-induced effects on neuronal survival, neuronal apoptosis and oxidative stress in the cerebral cortex of MCAO rats. **A**/**B** Nissl staining of neuron survival in each group after PVT1 overexpression and miR-24-3p up-regulation (× 200; scale bar = 50 μm, n = 8 rats per group); arrows indicated Nissl bodies; **C**/**D** TUNEL staining of neuronal apoptosis in each group after PVT1 overexpression and miR-24-3p up-regulation (× 200; scale bar = 50 μm, n = 8 rats per group; arrows indicated TUNEL-positive cells); **E** Oxidative stress indicators in the cortex of rats in each group after PVT1 overexpression and miR-24-3p up-regulation (n = 8 rats per group); ^^^*P* < 0.05 compared with the Lenti-NC group; ^$^*P* < 0.05 compared with the Lenti-PVT1 group

Nissl staining results depicted that in contrast to the Lenti-NC group, the Lenti-PVT1 group implied with largely dissolved Nissl bodies, lightly stained cytoplasm and reduced number of Nissl bodies (*P* < 0.05). In comparison with the Lenti-PVT1 group, the Lenti-PVT1 + Lenti-miR-24-3p group demonstrated darkly stained cytoplasm, increased number of Nissl bodies (*P* < 0.05) (Fig. 6A, B).

TUNEL staining manifested that versus the Lenti-NC group, increased number of TUNEL-positive neurons was tested in the the Lenti-PVT1 group; by comparing with the Lenti-PVT1 group, the Lenti-PVT1 + Lenti-miR-24-3p group exhibited reduced TUNEL-positive neurons (all *P* < 0.05) (Fig. 6C, D).

The levels of oxidative stress indicators in the cerebral cortex exhibited that by comparison with the Lenti-NC group, MDA content elevated while SOD and GSH activities diminished in the Lenti-PVT1 group (all *P* < 0.05). On the contrary, decreased MDA content, and enhanced SOD and GSH activities presented in the Lenti-PVT1 + Lenti-miR-24-3p group rather than the Lenti-PVT1 group (all *P* < 0.05) (Fig. 6E).

### SOX2 regulates STAT3 expression via activating PVT1 to sponge miR-24-3p

**Fig. 7:**
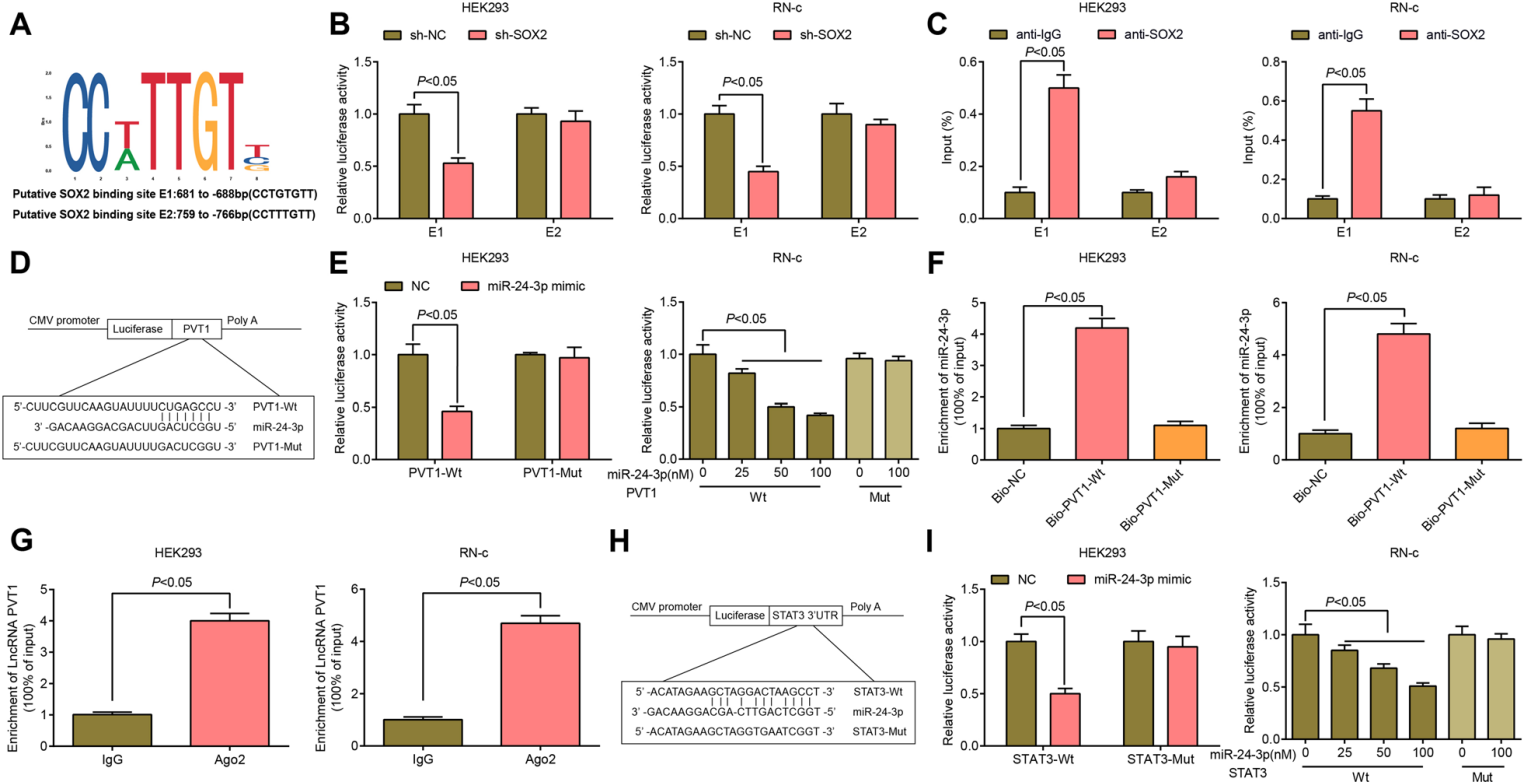
SOX2 regulates STAT3 expression via activating PVT1 to sponge miR-24-3p. **A** Bioinformatics website prediction of the binding site of SOX2 and PVT1 promoter; **B** Dual luciferase reporter gene assay of validation of the binding relationship between SOX2 and PVT1 in HEK293 cells and RN-c cells; **C** ChIP-qPCR assays of validation of the binding relationship between SOX2 and PVT1 in HEK293 cells and RN-c cells; **D** The surmised binding sites for interaction between PVT1 and miR-24-3p; **E** Dual luciferase reporter gene assay of verification of the binding of PVT1 and miR-24-3p in HEK293 cells and RN-c cells; **F** RNA-pull down assay of verification of the binding relationship between PVT1 and miR-24-3p in HEK293 cells and RN-c cells; **G** RIP assay of verification of the binding relationship between PVT1 and miR-24-3p in HEK293 cells and RN-c cells; **H** The surmised binding sites for correlation between miR-24-3p and STAT3; **I** Dual luciferase reporter gene assay of validation of the targeting relationship between miR-24-3p and STAT3 in HEK293 cells and RN-c cells; Repetition = 3

To explore the upstream regulatory mechanism of PVT1, JASPAR database (http://jaspar.genereg.net/) was used to screen possible transcriptional factors in the promoter of PVT1. It was found that SOX2 was bound to the PVT1 promoter, including two binding sites (TFBS; E1 and E2) (Fig. 7A). To further determine the specific binding site, a luciferase reporter vector containing two binding sites (E1, 6813 to 688 bp; E2, 759 to 766 bp) was constructed and inserted into the pGL3 vector (Promega). The dual luciferase reporter gene assay identified that sh-SOX2 reduced the luciferase activity in the E1 region of HEK293 cells and RN-c cells (Fig. 7B). ChIP assay was performed with the results showing that SOX2 could bind to the E1 site of PVT1 (Fig. 7C). Taken together, SOX2 was a direct upstream inducer of PVT1.

Through the prediction website (http://starbase.sysu.edu.cn/), it was found that PVT1 could bind with miR-24-3p (Fig. 7D), which was further verified by the dual luciferase reporter gene assay (Fig. 7E). After co-transfection with miR-24-3p mimic, no difference was found in the luciferase activity of pmirGLO-PVT1-Mut, but the luciferase activity of pmirGLO-PVT1-Wt was decreased (*P* < 0.05), indicating that miR-24-3p could specifically bind to PVT1. The outcome of RNA pull-down assay exhibited that (Fig. 7F) versus the Bio-NC group, the enrichment of miR-24-3p in the Bio-PVT1-Wt group increased (*P* < 0.05), while that of miR-24-3p in the Bio-PVT1-Mut group had no difference (*P* > 0.05). RIP analysis showed that compared with IgG, Ago2 recruited more PVT1 (*P* < 0.05) (Fig. 7G). The results indicated that PVT1 interacts with miR-24-3p.

The bioinformatics website (http://cm.jefferson.edu/rna22/Interactive) predicted that miR-24-3p had a targeted relationship with STAT3 (Fig. 7H) which was further verified by dual luciferase reporter gene assay (Fig. 7I). After transfection with STAT3-Wt and miR-24-3p mimic, the relative luciferase activity of HEK293 cells or RN-c cells was impaired (*P* < 0.05) while luciferase activity was not different with co-transfection of STAT3-Mut and miR-24-3p mimic (*P* > 0.05), implying that STAT3 was a direct target gene of miR-24-3p.

## Discussion

IS refers to a common neurological complication of infective endocarditis (Bettencourt and Ferro 2020). A study has outlined that suppression of SOX2 after MCAO alleviates neurobehavioral functional improvement in IS (Zhao et al. 2018). However, the more specific mechanisms of SOX2, together with PVT1, miR-24-3p and STAT3 should be discussed deeply. Hence, this study is practiced with the results concluding that SOX2 suppression improved IS via PVT1/miR-24-3p/STAT3 axis.

To begin with, this study has illustrated that SOX2, PVT1 and STAT3 were highly expressed while miR-24-3p was poorly expressed in brain tissues in MCAO rats. Actually, SOX2 is reported to be elevated in hypoxia injury (Han et al. 2017). Similarly, an increment is indicated in the expression of SOX2 in rental ischemia reperfusion injury (Rogers et al. 2016). In the light of the role of PVT1, there are studies implying that overexpressed PVT1 shows up in glioma and diffuse gliomas (Fu et al. 2018; Zou et al. 2017). Moreover, the expression of PVT1 is also upregulated in epileptic rats (Zhao et al. 2019). Mechanically, the reduction of miR-24-3p expression is presented in mice with hepatic ischemia and reperfusion injury (Shen et al. 2020). Furthermore, a prior study has highlighted the lower expression of miR-24-3p in cerebral ischemia-reperfusion injury (Kuai et al. 2021). As to the expression of STAT3, there has been a study depicting that STAT3 is activated in middle cerebral artery occlusion and reperfusion (Nakagawa and Aruga 2019). Anyway, our study findings are to some degree in consistent with previous studies.

Based on the findings of SOX2, PVT1, STAT3 and miR-24-3p expression, we have conducted a series of assays to decipher their roles in the progression of IS. The study has indicated that down-regulation of SOX2 or PVT1 improved brain injury in MCAO rats as reflected by neuronal apoptosis and oxidative stress restriction, brain water content reduction, and neurological deficit improvements. Depletion of PVT1 is evidenced to promote cell apoptosis in multiple myeloma (Zhang et al. 2020). Currently, a reduction of hsa_circ_PVT1 functionally stimulates cell apoptosis in glioblastoma multiforme (Chi et al. 2020). Besides that, depleted PVT1 is documented to serve as a suppressor of cardiomyocyte apoptosis in doxorubicin-induced cardiotoxicity (Zhan et al. 2020). Drawn from an advanced study, it is known that restoration of SOX2 enhances mitochondrial oxidative stress in corneal endothelial cells (Chang et al. 2018). Intriguingly, accelerated cell apoptosis is ascribed to silencing of SOX2 in Ewing’s sarcoma (Ren et al. 2016). Moreover, the depletion of SOX2 contributes to notable cell apoptosis in lung cancer (Chou et al. 2013). In conclusion, silencing of both PVT1 and SOX2 is a positive and active actor in diseases. Additionally, this study has also implied the protective role of up-regulated miR-24-3p in IS as evidenced by its ability to abolish PVT1 overexpression-induced brain injury in MCAO rats. In fact, miR-24-3p up-regulation could suppress apoptosis of cardiomyocytes conditioned to stimulated ischemia/reperfusion (Xiao et al. 2018). Moreover, up-regulated miR-24-3p is indicated to improve oxygen-glucose deprivation/reperfusion-induced neuron cell damage and oxidative stress (Di et al. 2021). Furthermore, it is surveyed that restoring miR-24-3p could limit apoptosis and inflammatory response of neurons in the course of cerebral ischemia-reperfusion injury (Kuai et al. 2021).

Lastly, another finding demonstrated in the present study is that SOX2 regulated STAT3 expression via activating PVT1 to sponge miR-24-3p. It is previously described that depletion of SOX2 is connected with the disturbance of the JAK2/STAT3 signaling pathway (Su et al. 2019). Moreover, the relationship between SOX2 and STAT3 has been discussed as reflected by SOX2 being the upstream of STAT3 pathway (Li et al. 2019). Besides that, it is also pointed out that overexpressed SOX2 promotes STAT3 activity (Huser et al. 2018). Wang et al. (2017) have discovered that induction of SOX2 can activate PVT1 in breast cancer. Many studies have demonstrated the functional role of PVT1 as a ceRNA to sponge miRs, such as miR-194-5p and miR-214-3p (Wang et al. 2020; Shang et al. 2019). However, nearly no study has explored the connection between PVT1 and miR-24-3p, and more investigations are supposed to fully develop the mechanism between the two factors.

## Conclusion

All in all, this study has discussed the mechanisms that down-regulating SOX2 improves IS via PVT1/miR-24-3p/STAT3 axis. This study may replenish the knowledge of pathogenesis and treatments of IS. However, more studies are required in a larger cohort for further development.

## Data Availability

Not applicable.

## Abbreviations

IS: Ischemic stroke
MCAO: Middle cerebral artery occlusion
PVT1: Plasmacytoma variant translocation 1
miR: MicroRNA
HIE: Hypoxic-ischemic encephalopathy
JAK: Janus kinase
circ: Circular
NC: Negative control
HE: Hematoxylin–eosin
TUNEL: Triphosphate-biotin nick end labeling
SOD: Superoxide dismutase
MDA: Malondialdehyde
GSH: Glutathione
ChIP: Chromatin immunoprecipitation
ANOVA: Analysis of variance
